# 

**Published:** 1886-02-20

**Authors:** 


### EDITORIALS AND MISCELLANEOUS.

DR. BALLINGER DEAD.—We regret to learn of the death of Dr. M. P. Ballinger, of Floyd County, Ga., which took place in January last.

DEATH OF DR. HARPER.—We are sorry to report the death of Dr. J. Harper, of Vicksburg, Miss., which occurred January 19, 1886. He had attained to the age of eighty-five years.

DR. N. S. DAVIS.—It is stated in one of our exchanges that Dr. N. S. Davis, editor of the Journal of the American Medical Association, was recently attacked with hemiplegia. At last account, he had in great measure recovered from the attack.

ABSTRACT AND NEW BOOKS.—The name of a neat and well conducted little journal, published at West Vermont, and edited by H. H. Howe, M. D. An important feature of this journal is the publication of a list of original articles as they occur in the various medical journals throughout the country.

SYNOPSIS OF PROCEEDINGS of the third annual meeting of the American Rhinological Association, held at Lexington, Ky., October 6, 7 and 8, 1885.

The above Association seems to be in an encouraging condition. A goodly number were in attendance. The president's address, by Dr. P. W. Logan, of Knoxville, is an interesting part of the pamphlet before us. We note approvingly his remark, that "Before practicing a specialty exclusively, we should have done general practice for several years; as a good general knowledge of disease is necessary to the management of special diseases." The discussions at the meeting were quite animated, particularly on the subject of Acute and Chronic Catarrh. There was a large accession of new members, among whom we notice the name of our colleague and esteemed fellow-citizen of Atlanta, Prof. A. G. Hobbs.

TRANSACTIONS OF THE TEXAS STATE MEDICAL ASSOCIATION, at its seventeenth annual session, held at Houston, April 21, 1885.

The above is a neatly gotten up book of 430 octavo pages.

The following are the officers elect for 1885–'86, viz: President—E. B. Becton, M. D., Sulphur Springs. Vice-Presidents—R. Rutherford, M. D., Houston; John C. Jones, M. D., Gonzales; Samuel R. Burroughs, M. D., Guy's Store. Secretary—W. J. Burt, M. D., Austin. Treasurer—J. Larendon, M. D., Houston.

The address of welcome was made by Hon. Charles Stewart in very appropriate terms, and responded to by President H. C. Ghent, M.D., who being the outgoing president, also made an able annual address.

Interesting papers were presented from the Sections on Medicine, Surgery, Larynæcology, Ophthalmology, Obstetrics, and Materia Medica, and Pathology. These, with a number of miscellaneous papers, constitute the body of the work.

The papers show remarkable enterprise, energy, and ability on the part of Texas physicians—to show our appreciation of which we copy in this issue of the RECORD the paper on Salex Nigra, by Dr. Paine, of Comanche.

A PALATABLE PREPARATION OF COCA.—The discovery of the anæsthetic power of cocaine has awakened renewed interest in the valuable therapeutic properties of Coca Erythroxylon, which is now in great demand.

Manufacturing pharmacists have not been slow to supply this demand, and the physician can now choose from a variety of preparations all claiming pre-eminent merit.

Owing to the inherent astringent and bitter constituents of the drug, however, it has been proved difficult to make a palatable preparation of coca which shall contain unchanged those principles of the drug on which depend its medicinal activity. This seems, however, to have been accomplished in the preparation we have before us—to wit, the Coca Cordial of Parke, Davis & Co.—which must commend itself to physicians wishing to prescribe a pure and agreeable tasting preparation of coca.

VICK'S FLORAL GUIDE FOR 1886, the pioneer seed annual of America, comes to us this year a real gem, not a dry list of hard botanical names, but over thirty pages of reading matter, among which are articles on Roses, House Plants, Cheap Greenhouse, Onion Culture, Mushrooms, Manures, Young Gardeners, and very interesting reading, followed by about 150 pages containing illustrations, descriptions and prices of seemingly everything the heart could desire in the line of Seeds, Plants, Bulbs, Potatoes, etc. It is a mystery how this firm can afford to publish, and really give away, this beautiful work of nearly 200 pages of finest paper, with hundreds of illustrations and two fine colored plates, all enclosed in an elegant cover. Any one desiring goods in this line cannot do better than to send 10 cents for the Floral Guide, to James Vick, Seedsman, Rochester, N. Y. Deduct the 10 cents from first order sent for seeds.

### *RECEIPTED.*

For 1886—W. P. Tercuit, J. E. Frippe, Jas. Ray, W. A. Sayle, to July; D. Maxwell; W. S. Bradford, to June; S. M. Hogan, S. L. Lockwood, T. M. Palmer, I. A. Nelms, Jno. Gerdine, T. L. Gipson, J. A. Ardry, to November; J. M. Bean, to October; L. S. Brownlee, E. H. M. Parrham, L. H. Hill, A. H. Askew, Jos. Underwood.

For 1885—J. S. Bushong, J. F. Earnest, W. H. Stewart, J. T. Kirkpatrick, to Sept. R. M. McBee, T. J. Charlton, E. Wheeler, to February, 1887; C. M. Griffin, to July, 1887.

